# Correction: Optimal paramedic numbers in resuscitation of patients with out-of-hospital cardiac arrest: A randomized controlled study in a simulation setting

**DOI:** 10.1371/journal.pone.0244400

**Published:** 2020-12-17

**Authors:** Bing Min Tsai, Jen-Tang Sun, Ming-Ju Hsieh, Yu-You Lin, Tsung-Chi Kao, Lee-Wei Chen, Matthew Huei-Ming Ma, Chiang Wen-Chu

The affiliation for the 1st author is incorrect. Bing Min Tsai is affiliated with both #1 and #5. Affiliation #5 is: Institute of Emergency and Critical Care Medicine, National Yang Ming University, Taipei City, Taiwan.
