# Supplementary material for: Optimal paramedic numbers in resuscitation of patients with out-of-hospital cardiac arrest: A randomized controlled study in a simulation setting
Source: PLoS One. 2020 Jul 7;15(7):e0235315. doi: 10.1371/journal.pone.0235315 (PMC7340314; doi:10.1371/journal.pone.0235315)
Supplement: S1 Appendix — (DOCX) [file pone.0235315.s001.docx]

**Appendix A**

The scenario environment and camera setup location
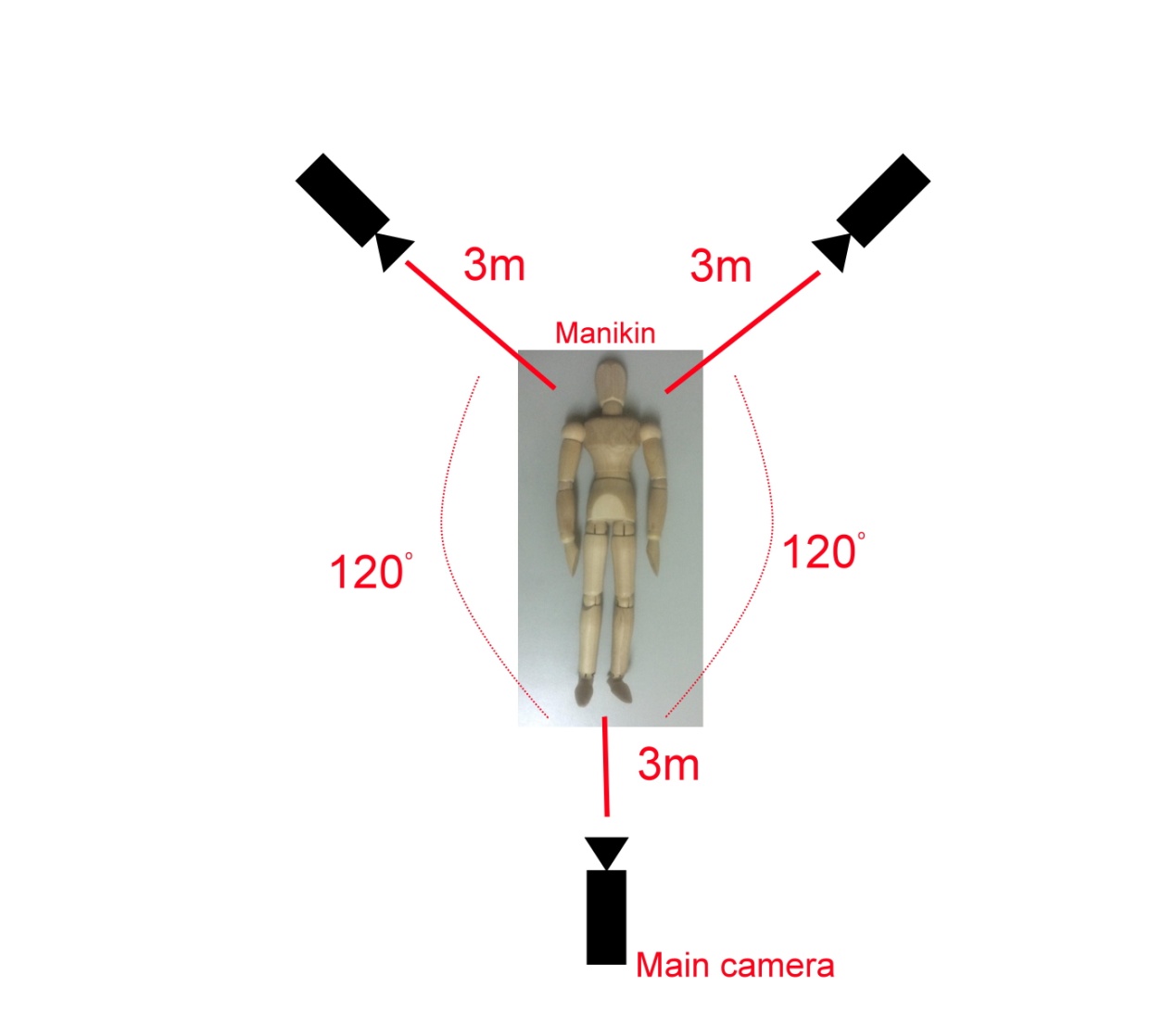


**Appendix B**

Teamwork performance evaluation scoring system

| Teamwork performance evaluation scoring system | | | | |
| --- | --- | --- | --- | --- |
| Leadership dedication | Rhythm management communications | Medication communications | CCF*monitoring | Score |
| Leadership is performed by a dedicated member **and** not distracted by primary interventions. |  |  |  | 4 |
| Leadership is performed by a dedicated member but is taskedto provide primary interventions. | Have proper response, ordered, and received a feedback (including rhythm, charge or not, **and** ongoing intervention) for **every**rhythm analyzed | Ordered and received a feedback (including name, dose, and route) for **every**medication administered |  | 3 |
| Command existed, but cannot tell which one is the main leader (multiple or invisible leader) | Have proper response, ordered, and received a feedback (including rhythm, charge or not,and ongoing intervention), but not every time or loss of some item | Ordered and received a feedback (including name, dose, and route) on some medications or loss item | Aware and responded correctly in every situation and potential (e.g. prolonged interruption for defibrillations) CPR interruptions | 2 |
| No leadership observed | No communication for every rhythm analyzed(except for clear before shocking) | No communication for every medication administered | Not aware or provided improper response to interruptions (sometimes or all the time) | 1 |

*CCF: chest compression fraction

**Appendix C**

The scatter plot of primary endpoint (overall chest compression fraction, CCF)

**
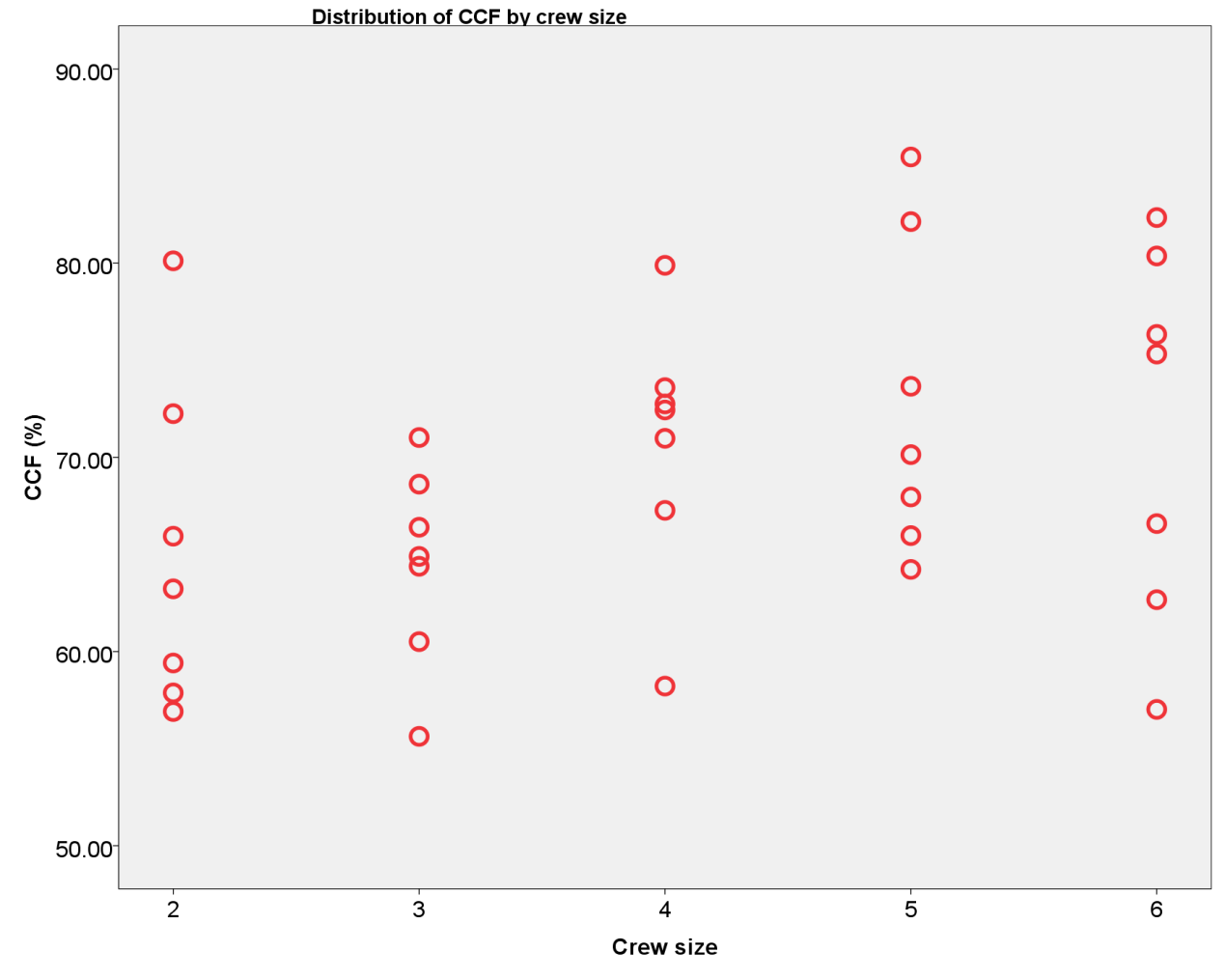
**

**Appendix D**

The scatter plot of chest compression fraction (CCF) during manual period (hand compression) and mechanical period (machine compression).

**
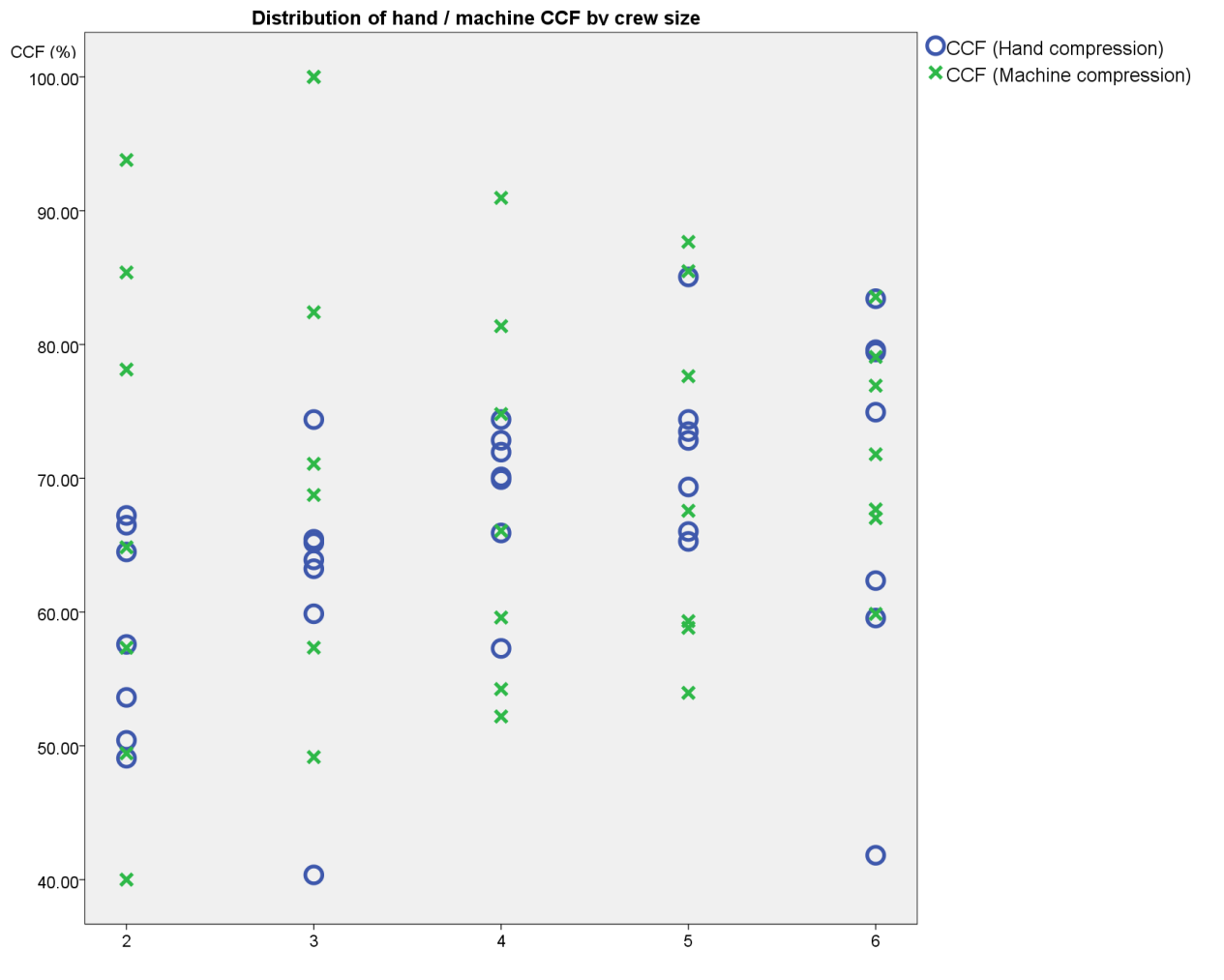

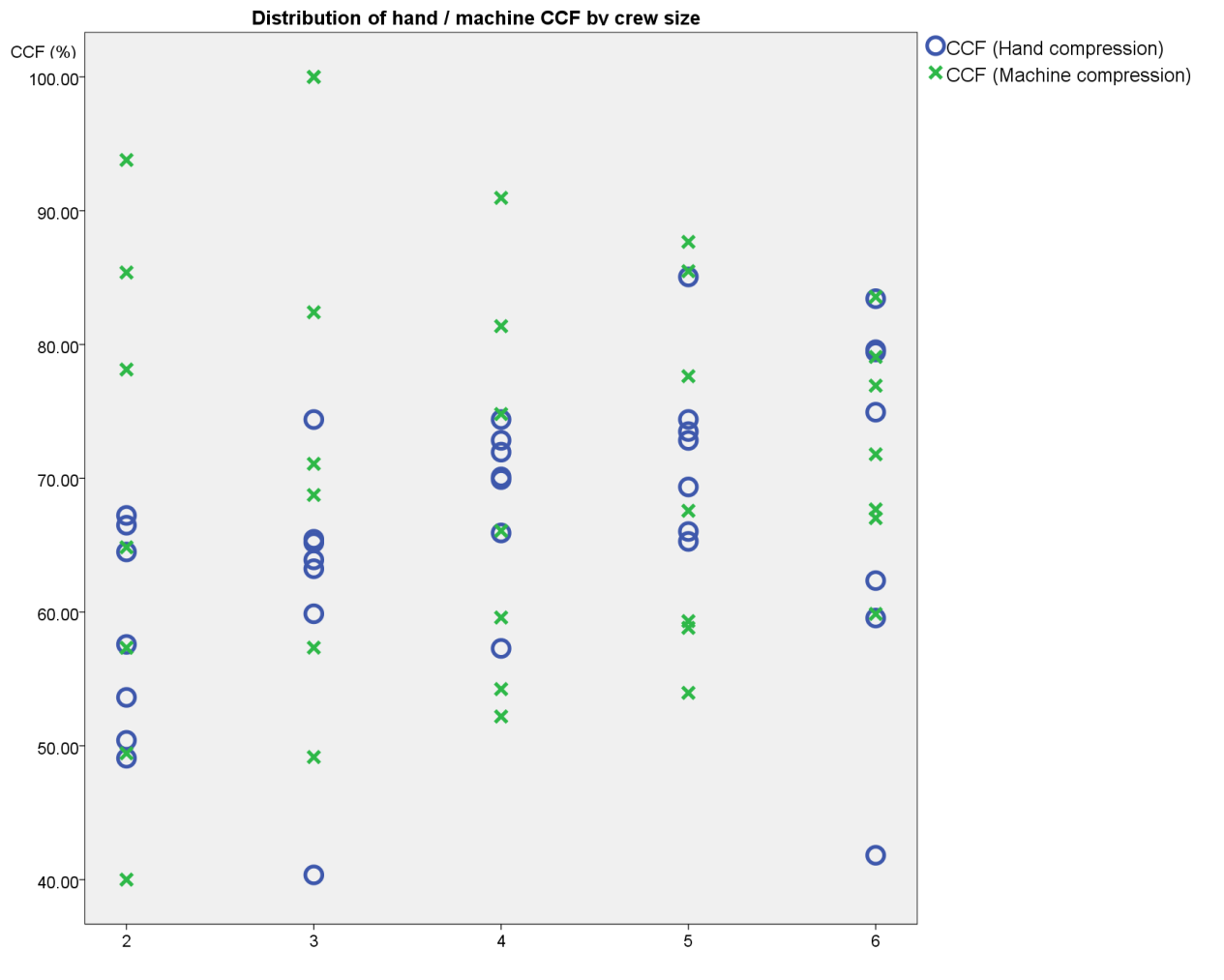
**
