# Supplementary material for: Optimal paramedic numbers in resuscitation of patients with out-of-hospital cardiac arrest: A randomized controlled study in a simulation setting
Source: PLoS One. 2020 Jul 7;15(7):e0235315. doi: 10.1371/journal.pone.0235315 (PMC7340314; doi:10.1371/journal.pone.0235315)
Supplement: Attachment — Submitted filename: Responses to the reviewers.docx [file pone.0235315.s002.docx]

PONE-D-20-07990

Optimal Paramedic Numbers in Resuscitation of Patients with Out-of-hospital Cardiac Arrest: A Randomized Controlled Study in a Simulation Setting

PLOS ONE

Dear Dr. WEN-CHU,

Thank you for submitting your manuscript to PLOS ONE. After careful consideration, we feel that it has merit but does not fully meet PLOS ONE’s publication criteria as it currently stands. Therefore, we invite you to submit a revised version of the manuscript that addresses the points raised during the review process.

We would appreciate receiving your revised manuscript by Jul 04 2020 11:59PM. When you are ready to submit your revision, log on to https://www.editorialmanager.com/pone/ and select the 'Submissions Needing Revision' folder to locate your manuscript file.

If you would like to make changes to your financial disclosure, please include your updated statement in your cover letter.

To enhance the reproducibility of your results, we recommend that if applicable you deposit your laboratory protocols in protocols.io, where a protocol can be assigned its own identifier (DOI) such that it can be cited independently in the future. For instructions see: http://journals.plos.org/plosone/s/submission-guidelines#loc-laboratory-protocols

Please include the following items when submitting your revised manuscript:

A rebuttal letter that responds to each point raised by the academic editor and reviewer(s). This letter should be uploaded as separate file and labeled 'Response to Reviewers'.

A marked-up copy of your manuscript that highlights changes made to the original version. This file should be uploaded as separate file and labeled 'Revised Manuscript with Track Changes'.

An unmarked version of your revised paper without tracked changes. This file should be uploaded as separate file and labeled 'Manuscript'.

Please note while forming your response, if your article is accepted, you may have the opportunity to make the peer review history publicly available. The record will include editor decision letters (with reviews) and your responses to reviewer comments. If eligible, we will contact you to opt in or out.

We look forward to receiving your revised manuscript.

Kind regards,

Corstiaan den Uil

Academic Editor

PLOS ONE

Journal requirements:

When submitting your revision, we need you to address these additional requirements:

1. Please ensure that your manuscript meets PLOS ONE's style requirements, including those for file naming. The PLOS ONE style templates can be found at http://www.plosone.org/attachments/PLOSOne_formatting_sample_main_body.pdf and http://www.plosone.org/attachments/PLOSOne_formatting_sample_title_authors_affiliations.pdf

2. Please include your tables as part of your main manuscript and remove the individual files. Please note that supplementary tables (should remain/ be uploaded) as separate "supporting information" files

3. We note you have included a table to which you do not refer in the text of your manuscript. Please ensure that you refer to Table 2 in your text; if accepted, production will need this reference to link the reader to the Table.

4. Please include captions for your Supporting Information files at the end of your manuscript, and update any in-text citations to match accordingly. Please see our Supporting Information guidelines for more information: http://journals.plos.org/plosone/s/supporting-information.

[Note: HTML markup is below. Please do not edit.]

Reviewers' comments:

Reviewer's Responses to Questions

Comments to the Author

1. Is the manuscript technically sound, and do the data support the conclusions?

The manuscript must describe a technically sound piece of scientific research with data that supports the conclusions. Experiments must have been conducted rigorously, with appropriate controls, replication, and sample sizes. The conclusions must be drawn appropriately based on the data presented.

Reviewer #1: Yes

Reviewer #2: Yes

2. Has the statistical analysis been performed appropriately and rigorously?

Reviewer #1: Yes

Reviewer #2: No

3. Have the authors made all data underlying the findings in their manuscript fully available?

The PLOS Data policy requires authors to make all data underlying the findings described in their manuscript fully available without restriction, with rare exception (please refer to the Data Availability Statement in the manuscript PDF file). The data should be provided as part of the manuscript or its supporting information, or deposited to a public repository. For example, in addition to summary statistics, the data points behind means, medians and variance measures should be available. If there are restrictions on publicly sharing data—e.g. participant privacy or use of data from a third party—those must be specified.

Reviewer #1: Yes

Reviewer #2: Yes

4. Is the manuscript presented in an intelligible fashion and written in standard English?

PLOS ONE does not copyedit accepted manuscripts, so the language in submitted articles must be clear, correct, and unambiguous. Any typographical or grammatical errors should be corrected at revision, so please note any specific errors here.

Reviewer #1: Yes

Reviewer #2: Yes

5. Review Comments to the Author

Please use the space provided to explain your answers to the questions above. You may also include additional comments for the author, including concerns about dual publication, research ethics, or publication ethics. (Please upload your review as an attachment if it exceeds 20,000 characters)

Reviewer #1: Thank you for giving me the opportunity to review your article. I could read this article with interest, however I felt some concern about your study.

The author mention the more crow reduce the time of intubation, and injection. However, there are many reports which do not recommend intubation and recommend compression only CPR. Therefore, this report has no worth for the investigators who believe such reports.

**Response from the authors:**

Thank you for your comments.

The effect of prehospital advanced life support (ALS), including endotracheal intubation and intravenous epinephrine on the outcome of patients with out-of-hospital cardiac arrest (OHCA), remained inconclusive in literature. However, there are many studies here in Taiwan showing the positive association between ALS and OHCA patient outcomes (reference 1, 2, 3 beneath), and that’s why we have to analyze the effect of crew size on these interventions. However, we do agree with your concern and will add it in the revised manuscript.

*Reference1, 2, 3*

1. *The Effect of Successful Intubation on Patient Outcomes after Out-of-Hospital Cardiac Arrest in Taipei. Chiang WC, Hsieh MJ, Chu HL, et al. Annals Emergency Medicine. 2018; 71(3):387-396.*
2. *The effect of the number and level of emergency medical technicians on patient outcomes following out of hospital cardiac arrest in Taipei. Sun JT, Chiang WC, Hsieh MJ, et al. Resuscitation. 2018;122:48-53*
3. *Prehospital intravenous epinephrine may boost survival of patients with traumatic cardiac arrest: a retrospective cohort study. Chiang WC, Chen SY, Ko PCI et al. Scandinavian Journal of Trauma, Resuscitation and Emergency Medicine 2015; 23:102-108.*

**Changes made (Please refer to the marked version):**

Page 14, Line 8:

Finally, this study examined the optimal crew size based on the CCF and prehospital ALS (i.e. endotracheal intubation and intravenous epinephrine). The results may not be applicable to those EMS systems without the recommendation of prehospital ALS in the field.

What kind of guideline did this study follow? RCA?

**Response from the authors:**

Thank you.

Paramedics here in Taiwan resuscitate the OHCA patients in the prehospital setting following the recommendations of ILCOR (International Liaison Committee on Resuscitation), but the timing of applying a mechanical CPR device during resuscitation has not been uniformly regulated in the EMS protocol of New Taipei City.

We have clarified this in the revised manuscript.

**Changes made (Please refer to the marked version):**

Page 9, Paragraph 2:

Paramedics here in Taiwan resuscitate the OHCA patients in the prehospital setting following the recommendations of ILCOR (International Liaison Committee on Resuscitation), but the timing of applying a mechanical CPR device during resuscitation has not been uniformly regulated in the EMS protocol of New Taipei City. Hence, the time for deployment of M-CPR device was decided by every team themselves.

In this study, the author recommend the best cure size is 5. However, transportation is also the important work for the emergency medical service. In practice, can 5 crows work in an ambulance?

**Response from the authors:**

The goal of this investigation aimed to help determine the best crew size for resuscitation of an OHCA patient in the field before ambulance transportation, thus we designed a simulation-based randomized controlled trial with cardiac arrest scenario equipped with widely adopted mechanical CPR to test different crew sizes. Based on the local EMS protocol, we will dispatch the 2nd crew to the scene in case of encountering an OHCA patient, but the transportation is still with only 2 paramedics in the ambulance patient compartment, not 5.

Thank you for your comments. We have clarified this in the revised manuscript.

**Changes made (Please refer to the marked version):**

Page 6, Line 17-18:

To help determine the best crew size for resuscitation of an OHCA patient in the field before ambulance transportation, we designed a simulation-based randomized controlled trial…

Reviewer #2: This study investigated the effect of paramedic crew size in the resuscitation of patients with out-of-hospital cardiac arrest (OHCA) remains inconclusive. The hypothesis was teams with a larger crew size have better resuscitation performance including chest compression fraction (CCF), advanced life support (ALS), and teamwork performance than those with a smaller crew size. The study was well designed with strong point as a randomized one.

Below are some comments:

1. The primary endpoint, chest compression fraction (CCF) is essentially continuous data with interval scale at the very least. And this has been used to power the study. However, the test statistics for the primary endpoint is based on non-parametric method, Kruskal-Wallis instead of 1-way ANOVA. Is there any particular reasons for using this non parametric approach? As it is commonly known, the use of non-parametric method may reduce the power to detect the intended meaningful difference stated in the hypothesis

**Response from the authors:**

Thank you for your comments. We used the Kruskal-Wallis method for our primary end point because our sample size is relatively small, only 7 outcome data in each crew-size group.

We have clarified this in the revised manuscript.

**Changes made (Please refer to the marked version):**

Page 11, line 12-14:

… the Kruskal-Wallis method was used to examine significance because of sample size limitation (i.e. only 7 outcome data in each crew-size group).

2. There is not any plot showing the distribution of the primary endpoint chest compression fraction (CCF) among the five groups. This is an important plot that will enable us to check a few things for example the data distribution and hence whether assumption on normality is fulfilled.

**Response from the authors:**

Thank you for your comments to make our manuscript more precise.

It is important to plot the distribution of the primary endpoint.

We’ve added scatter plots for primary and secondary outcomes in the revised manuscript.

**Changes made (Please refer to the marked version):**

Page 12, Line 16: The scatter plot of CCF data was shown in Appendix C and D.

Appendix Figure C: The scatter plot of primary endpoint (overall chest compression fraction, CCF)

Appendix Figure D: The scatter plot of chest compression fraction (CCF) during manual period (hand compression) and mechanical period (machine compression).

3. In the statistical analysis section, it is mentioned about ‘For all non-parametric continuous data, … ‘. It is not defined what it means by non—parametric continuous data. What is the criteria for non-parametric?

**Response from the authors:**

Thank you for reminding us of the misused statistical term.

We have made a correction.

The criteria for non-parametric in this study was its sample size limitation. Because there was only 7 outcome data in each crew-size group, the assumption on normality is not fulfilled.

**Changes made (Please refer to the marked version):**

Page11, line11:

... ~~all non-parametric continuous data~~ The Kruskal-Wallis method was used to examine significance for sample size limitation (i.e. only 7 outcome data in each crew-size group), not fulfilling the assumption of normality.

4. For the Kaplan-Meier method used to compare the time-to-accomplishment of ALS intervention (i.e. first-dose of epinephrine and success of endotracheal intubation), there are 5 groups to compare yet only a single p-value is reported there. What exactly this single p-value represent? Have you done any further analyses to do pairwise comparisons of the KM curves? Have any adjustment of the p-values made?

**Response from the authors:**

Thank you for your comments.

The single p-value reported here is the overall p-value. Statistically significant results indicate that not all of the group means are equal.

Per your suggestions, we have consulted the staff of National Taiwan University Hospital-Statistical Consulting Unit (NTUH-SCU), and performed post hoc tests to do pairwise comparisons with the adjusted level of the p-value of the KM curves. The results were reported on the revised figure 3A&3B.

Thank you for making our manuscript more informative to the readers.

**Changes made (Please refer to the marked version):**

Page 12, line 1:

Post hoc tests were performed to do pairwise comparisons for the primary endpoint (CCF) and Kaplan-Meier curves, with the adjusted level of the p-value for multiple comparisons.

Figure. 3A & 3B (figure tag: results of overall p-value and post hoc test)

5. In the same question as in (4) is also for time to finish intubation

**Response from the authors:**

Thank you for the suggestions.

**Changes made (Please refer to the marked version):**

Page 12, line 1:

Post hoc tests were performed to do pairwise comparisons for the primary endpoint (CCF) and Kaplan-Meier curves, with the adjusted level of the p-value for multiple comparisons.

Figure. 3A & 3B (figure tag: results of overall p-value and post hoc test)

6. For the primary endpoint CCF, how is the result if using ANOVA with the obtained/observed CCF instead of the ranks? With this approach, is it possible to do further analyses of pairwise comparisons between groups?

**Response from the authors:**

Thank you for your comments. We have made changes accordingly.

Per your suggestions, we performed post hoc tests with the CCF.

The results were reported on the revised Table 2.

Thank you for making our manuscript more informative to the readers.

**Changes made (Please refer to the marked version):**

Revised Table. 2 (with a new column “post hoc tests”)

6. PLOS authors have the option to publish the peer review history of their article (what does this mean?). If published, this will include your full peer review and any attached files.

If you choose “no”, your identity will remain anonymous but your review may still be made public.

Do you want your identity to be public for this peer review? For information about this choice, including consent withdrawal, please see our Privacy Policy.

Reviewer #1: Yes: Shuichi Hagiwara

Reviewer #2: No

[NOTE: If reviewer comments were submitted as an attachment file, they will be attached to this email and accessible via the submission site. Please log into your account, locate the manuscript record, and check for the action link "View Attachments". If this link does not appear, there are no attachment files to be viewed.]

While revising your submission, please upload your figure files to the Preflight Analysis and Conversion Engine (PACE) digital diagnostic tool, https://pacev2.apexcovantage.com/. PACE helps ensure that figures meet PLOS requirements. To use PACE, you must first register as a user. Registration is free. Then, login and navigate to the UPLOAD tab, where you will find detailed instructions on how to use the tool. If you encounter any issues or have any questions when using PACE, please email us at figures@plos.org. Please note that Supporting Information files do not need this step.
